# Effectiveness of acupuncture combined with auricular acupressure in the treatment of postoperative ileus: a study protocol for a randomized controlled trial

**DOI:** 10.3389/fsurg.2024.1349975

**Published:** 2024-06-03

**Authors:** Ruoyun Lyu, Zonglin Wen, Rong Huang, Zhiling Yang, Yingqun Chen

**Affiliations:** ^1^ Department of Traditional Chinese Medicine, Shanghai Tenth People’s Hospital, Shanghai, China; ^2^ Department of Tuina, Shuguang Hospital Affiliated to Shanghai University of Traditional Chinese Medicine, Shanghai, China

**Keywords:** postoperative ileus, acupuncture, auricular acupressure, ERAS, protocol

## Abstract

**Background:**

About one-third of patients experience postoperative ileus (POI) after abdominal surgery, which can cause various complications and has not been treated well in clinical practice. The comprehensive treatment offered by traditional Chinese medicine may be a good choice for promoting intestinal mobility. Therefore, the aim of this study protocol is to observe the effectiveness of acupuncture combined with auricular acupressure in decreasing the incidence and related symptoms of POI.

**Methods:**

This is a single-center, assessor-blinded, randomized controlled trial. A total of 160 participants are supposed to recruit at Shanghai Tenth People's Hospital and randomly divided into two parallel groups in a 1:1 ratio. The intervention group are planned to receive manual acupuncture combined with auricular acupressure, while the control group are planned to receive regular enhanced recovery after surgery treatment. The primary outcome is the time to first defecation and first flatus after surgery. The secondary outcomes include the length of postoperative hospital stay, intensity of postoperative abdominal pain and distension, severity of postoperative nausea and vomiting, time to tolerate diet, inflammatory index, and incidence of prolonged postoperative ileus.

**Discussion:**

The results of this research will provide substantial evidence regarding the efficacy of comprehensive traditional Chinese treatment, specifically auricular acupressure and manual acupuncture, in treating and preventing POI.

**Trial registration:**

ClinicalTrials.gov, Identifier: ChiCTR2300075983, registered on September 21, 2023.

## Background

Postoperative ileus (POI) is an inevitable complication after abdominal surgery, which reflects a deceleration or complete cessation of bowel movements (1). Patients may present with symptoms such as abdominal pain and distension, vomiting, anal defecation, and defecation disorders. Research shows that POI occurring within 3 days for laparoscopic surgery or occurring within 5 days for open surgery is considered physiological; however, if it continues beyond these time frames, it can be considered pathological (2). POI is closely linked with negative factors, including an extended hospital stay, a higher likelihood of hospital-acquired infections or venous thromboembolism, and an increased rate of 30-day readmissions (3–5). The incidence of pathological POI can reach up to 10%–30% (6, 7) in abdominal surgery, which has become a current topic of concern in clinics.

To reduce the occurrence of POI, the conception of the enhanced recovery after surgery (ERAS) protocol has been implemented throughout the perioperative period (8). Many strategies have been supposed to promote bowel movement after surgery; however, only a few of them show a beneficial effect, such as μ-opioid-receptor antagonists, non-steroidal anti-inflammatory drugs (NSAIDS), laparoscopic surgery, and mid-thoracic epidural anesthesia (9–11). More strategies are still under exploration.

The inflammatory phase induced by abdominal surgery greatly impacts gastrointestinal dynamics and is therefore regarded as the main period of clinical prevention and treatment of POI (12). Several systematic reviews of randomized controlled trials (RCTs) on the effect of acupuncture and related therapies on bowel function after surgery show that acupuncture has a positive effect on bowel movement, including improvement of first flatus, first defecation, and first bowel sound (13–15). Manual acupuncture (MA) studies have shown anti-inflammatory effects in the digestive system through vagus nerve activation, TLR4/MyD88/NF-κB signaling, macrophage polarization, the mitogen-activated protein kinase (MAPK) signaling pathway, and the cholinergic anti-inflammatory pathway (16–18), which can be considered as the possible mechanisms of acupuncture in the treatment of POI. Another study reveals that acupuncture of hindlimb regions inhibits the expression of GABAA receptor in DMV neurons, which excites the vagal nerve and, in turn, suppresses inflammation via activation of the α7nAChR-mediated JAK2/STAT3 signaling pathway of POI (19). Auricular acupressure (AA) is another popular complementary approach for postoperative gastrointestinal dysfunction in abdominal surgery, which is non-invasive and has been approved to decrease the incidence of postoperative constipation (20). In clinical practice, these two treatment methods are always combined for simultaneous use; however, few research studies have focused on the comprehensive effect of their combined application in the treatment of POI.

Hence, this single-center, randomized controlled trial is designed to observe the effectiveness of acupuncture combined with auricular acupressure in decreasing the incidence and related symptoms of POI.

## Study design

**Figure 1:**
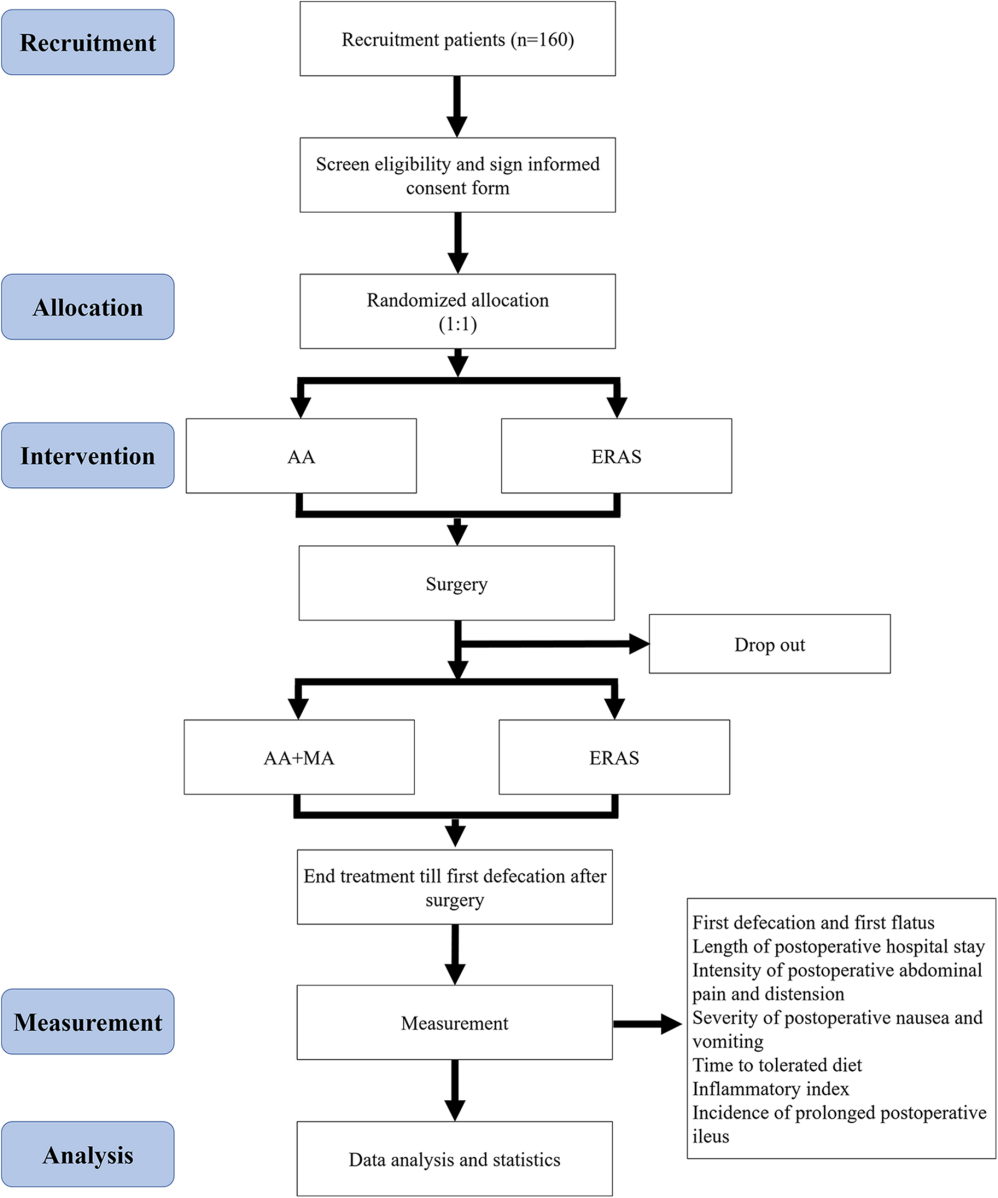
Flowchart of research.

The single-center, controlled, assessor-blinded, randomized trial is being executed in Shanghai Tenth People's Hospital from July 2023 and will continue until June 2026. The protocol is described in accordance with the Consolidated Standards of Reporting Trials (CONSORT) (21) guidelines and the Standards for Reporting Interventions in Controlled Trials of Acupuncture (STRICTA) (22). The study is registered at the Chinese Clinical Trial Registry (Identifier: ChiCTR2300075983) and is approved by the Ethics Committee of Shanghai Tenth People's Hospital (No. SHSY-IEC-5.0/23K82/P01). A total of 160 participants with elective laparoscopic surgery will be randomly assigned into an intervention group and a control group with a ratio of 1:1. Details of trial design and analysis plans are presented in Figure 1 and Table 1.

**Table 1:** Details of the study period.

	Study period				
	Visit 1	Visit 2	Visit 3	Visit 4	Visit 5
Time node	−7 to 0 day	0 day	3 days before surgery	Day 1–3 after surgery	Discharged from the hospital
Enrollment					
Informed consent	●				
General information	●				
Medical history	●				
Randomization		●			
Intervention					
AA + MA			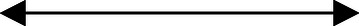		
ERAS			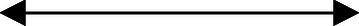		
Assessment					
First defecation				●	
First flatus				●	
Length of postoperative hospital stay					●
Intensity of abdominal pain and distension				●	●
Severity of PNOV				●	●
Time to tolerate diet				●	●
Inflammatory index		●		●	●
Incidence of PPOI					●
Safety observation			●	●	●
Adverse event record			●	●	●
Obedience evaluation					●

## Recruitment

Patients in the abdominal surgery department of Shanghai Tenth People's Hospital will be diagnosed by a surgeon. All eligible participants will be explained the trial in detail by researchers and will be required to sign informed consent prior to participation. They will also have the right to withdraw from the trial anytime.

## Eligibility criteria

### Inclusion criteria

(1) Age ranging from 18 to 80 years;
(2) Having upper gastrointestinal disease who will receive elective laparoscopic surgery, including benign hepatobiliary diseases, pancreatic diseases, gastric diseases, and malignant non-metastatic tumors;
(3) Willing to sign the informed consent form and participate in the clinical trial.

### Exclusion criteria

(1) A history of serious diseases such as cardiovascular, cerebrovascular, liver, kidney, hematopoietic, digestive system, or mental illness;
(2) A history of surgery in the stomach or intestine;
(3) Having taken drugs for inhibiting gastrointestinal motility for over 2 weeks in the last 3 months;
(4) Having received preoperative chemotherapy or radiotherapy;
(5) Having received relevant treatments that may influence the effect of the study;
(6) Unable to understand and fill out the scales related to the research.

## Randomization and blinding

The randomized program is developed using the Statistical Package for Social Sciences (SPSS) Ver. 24.0 (IBM Inc., New York, USA), and the opaque random envelope is used by the assistant to achieve the allocation concealment. The block grouping method is used to achieve randomization and set the length of the block group as 4. The random envelope is marked with the serial number of the group, and the group number is printed on the envelope. The envelopes are sequentially opened according to the time sequence of the included cases, and the control group and the intervention group are divided according to the groups in the envelope.

The participants and the acupuncturist will not be blinded because of the specialization of acupuncture manipulation. However, the data analysis and efficacy assessment will be performed by the research assistant and blinded to decrease analysis bias.

## Interventions

### Intervention group: MA combined with AA

MA and auricular acupressure procedures of the intervention group will be administered by licensed acupuncturists with 5–12 years of education in acupuncture and at least 3 years of clinical experience. They will all undergo unified and standardized training before trial initiation.

**Figure 2:**
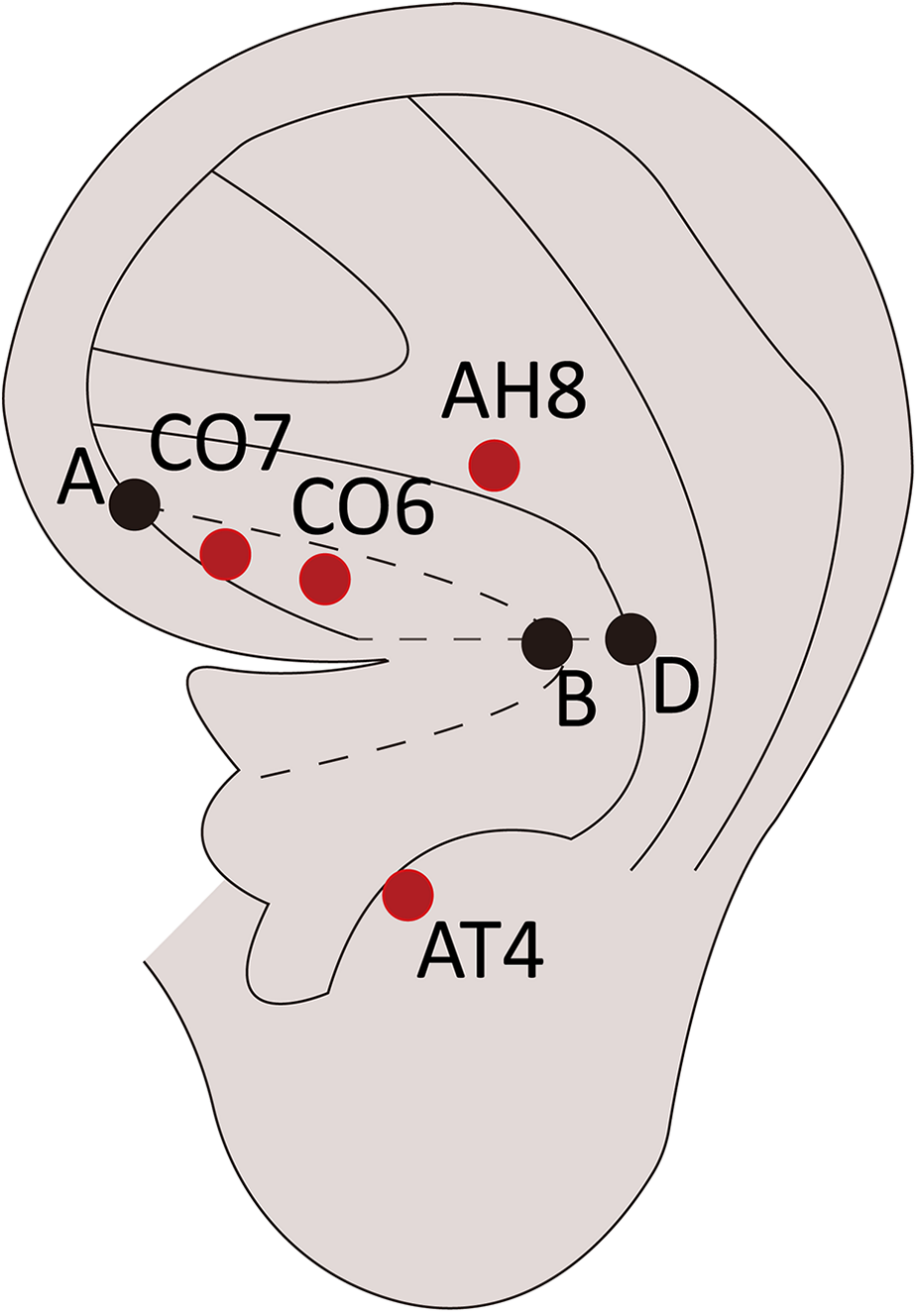
Location of auricular acupoints.

The intervention group will accept AA at the small intestine (CO6), large intestine (CO7), abdomen (AH8), and sub-cortex (AT4) according to the *Nomenclature and Location of Auricular Points* (Table 2 and Figure 2). Vaccaria seeds (Wang-Bu-Liu-Xing) (40 mm × 40 mm) (Changshu Shenling Medical Products Factory Co., Ltd., Changshu, China) will be embedded on the surface of auricular points and maintained between the two treatments. Two ears will accept AA alternately once daily. Participants will be suggested to press each point gently for 1 min thrice daily. The treatment will start 3 days before elective laparoscopic surgery and continue until the first defecation after surgery.

**Table 2:** Location of auricular acupoints.

Auricular acupoints	Locations
Small intestine (CO6)	At the intermediate 1/3 of the region between the helix crus and Line AB^a^
Large intestine (CO7)	At the anterior 1/3 of the region between the helix crus and Line AB
Abdomen (AH8)	On the upper 2/5 of the body of the antihelix crus
Sub-cortex (AT4)	On the medial side of the antitragus

Point A: At the medial edge of the helix at the junction between the middle and upper 1/3 of the line from the notch of the helix crus and the inferior edge of the inferior antihelix crus. Point B: At the junction of the middle and posterior 1/3 of the line extending from the end of the helix crus to Point D. Point D: At a level line drawn from the end of the helix crus crossing the concha edge of the antihelix.

^a^ Line AB: A curved line that extends from Point A to Point B and mirrors the inferior edge of the helix crus.

**Figure 3:**
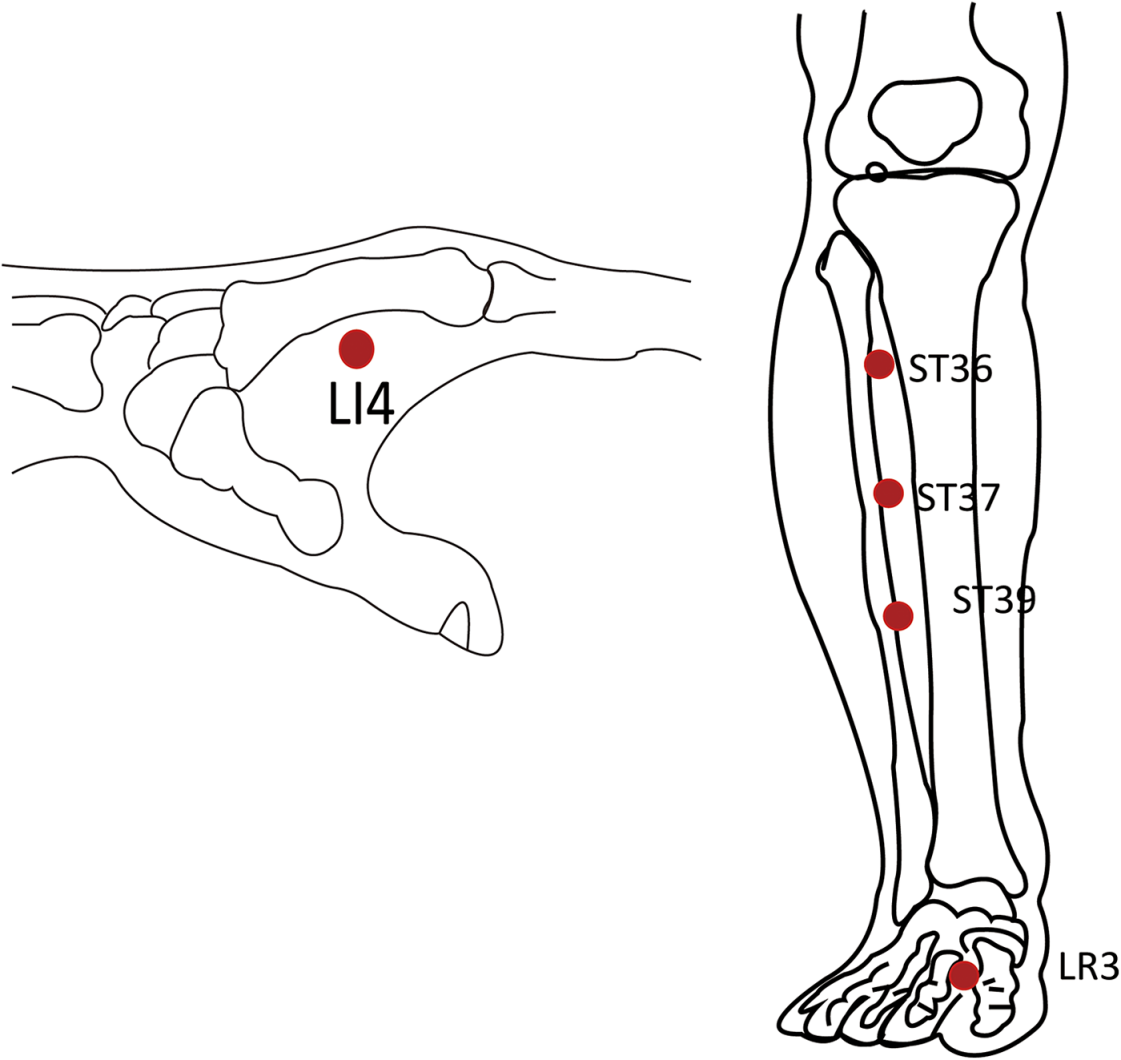
Location of acupoints.

The intervention group will also accept MA at *Zusanli* (ST36), *Shangjuxu* (ST37), *Xiajuxu* (ST39), *Hegu* (LI4), and *Taichong* (LR3) according to *Acupuncture and Moxibustion* (23) (Table 3 and Figure 3). A single-use stainless steel needle (0.25 mm × 40 mm) (Suzhou Medical Products Factory Co., Ltd., Suzhou, China) will be used, and Deqi sensation (a sensation of sourness, numbness, or distension) should be achieved during the process. The needle should be retained for 20 min and then withdrawn quickly. Patients will receive MA once a day for consecutive days after surgery until the first defecation after surgery.

**Table 3:** Location of acupoints.

Acupoints	Locations
Zusanli (ST36)	On the lateral aspect of the leg, 3 cun inferior to Dubi (ST35), on the line connecting Dubi^a^ (ST35) and Jiexi^b^ (ST41)
Shangjuxu (ST37)	On the lateral aspect of the leg, 6 cun inferior to Dubi (ST35), on the line connecting Dubi(ST35) and Jiexi(ST41)
Xiajuxu (ST39)	On the lateral aspect of the leg, 9 cun inferior to Dubi (ST35), on the line connecting Dubi (ST35) and Jiexi (ST41)
Hegu (LI4)	On the dorsum of the hand, radial to the midpoint of the second metacarpal bone
Taichong (LR3)	On the dorsum of the foot, between the first and second metatarsal bones, in the depression anterior to the junction of the bases of the two bones, or over the dorsalis pedis artery

^a^ Dubi (ST35): On the anterior aspect of the knee, in the depression lateral to the patellar ligament.

^b^ Jiexi (ST41): On the anterior region of the ankle, in the depression at the center of the front surface of the ankle, between the extensor halluces longus tendon and the extensor digitorum longus tendon.

### Control group: ERAS

The control group will receive regular treatment of ERAS 3 days before surgery because patients are always admitted to the hospital several days earlier for standard preoperative blood testing and bowel preparation, including taking oral anti-inflammatory drugs, having liquid food 72 h before the operation, accepting enema 24 h before the operation, using intravenous antibiotics to preventing infection, and so on. In addition, the control group will be initiated on a liquid diet within 24 h after surgery to stimulate bowel movement.

Patients in both groups will receive the same postoperative procedures in mobilization to avoid bias in evaluating the onset of POI, including getting out of bed within 24 h after surgery and walking three times a day for a distance of 50–100 m after 48 h.

## Outcome measures

### Primary outcome measures

The primary outcomes are the time to first defecation and first flatus, which is regarded as a bowel function indicator. This time is calculated from the end of laparoscopic surgery and will be recorded by nurses with accuracy to the hour.

### Secondary outcome measures

#### Length of postoperative hospital stay

The length of postoperative hospital stay is an important index used to determine the clinical effect of treatment, which is relatively objective and intuitive (24–26).

#### Intensity of postoperative abdominal pain and distension

The abdominal pain will be assessed by the visual analog scale (VAS), which has been proven less vulnerable to confounding factors and ceiling effects than other pain scales (27–29). The intensity of abdominal distension can be divided into four grades: no symptoms, mild, moderate, and severe. Both indexes will be obtained 1 day after surgery and at the time of discharge.

#### Severity of postoperative nausea and vomiting

The simplified postoperative nausea and vomiting (PONV) impact scale, which consists of two questions, will be used to evaluate postoperative nausea and vomiting (30). Clinical PONV can be defined when the impact scale score is more than 5.

#### Time to tolerate diet (liquid or semi-liquid food)

Postoperative ileus can result in delays in time to mobilization and resumption of oral intake, which may further affect patient's satisfaction and wound recovery (31). Therefore, the time to tolerate diet will be recorded as a reference.

#### Inflammatory index

The inflammatory index includes white blood cell (WBC) count and the levels of C-reactive protein (CRP), interleukin-6 (IL-6), procalcitonin (PCT), and tumor necrosis factor-α (TNF-α), which will be obtained at baseline, 1 day after surgery, and at the time of discharge.

#### Incidence of prolonged postoperative ileus

Prolonged postoperative ileus (PPOI) can be diagnosed when at least two of the following symptoms occur after 96 h postoperatively: nausea or vomiting, inability to tolerate any oral diet over the last 24 h, abdominal bloating and distension, or radiological confirmation (32).

## Safety and acupuncture-related adverse events

Acupuncture may cause several adverse reactions or adverse events (33), such as bleeding, pain, dizziness, syncope, needle-sticking, and the like. Therefore, clinical safety will be monitored throughout the trial, and all adverse events will be reported. The patients with adverse reactions may receive relevant treatment, and the possible reasons for adverse events will be discussed to avoid the next occurrence. The number and type of all adverse reactions and events will be summarized during the final safety assessment. The participants will be withdrawn from the study if they experience severe adverse events, and relevant information will be immediately reported to the primary investigator.

## Data and safety monitoring board

Before the start of the trial, all members of this study will have special training on clinical trial operation and quality control. A data and safety monitoring board will also be established to ensure data safety and protect the rights and health of all the participants. This independent advisory group will check the data and monitor the execution of scientific and ethical standards of the RCT once a week. Participants’ information will be saved in the specified file folder and provided only with the permission of the data and safety monitoring board.

## Sample size calculation

Our primary outcome is the time to first defecation. The unpublished experimental data on the effects of AA combined with MA, based on a 2-week prior pilot experiment, were used for the sample size calculation. The time to first defecation is 77.8 ± 15.6 h in the intervention group and 85.1 ± 13.35 h in the control group. We chose the two-sample *t*-test model with a two-tailed test, and the relevant calculating parameters are a significance level of 0.05, a power of 0.80, and an allocation ratio of 1:1. The required number of participants per group is 72. Considering a dropout rate of 10%, the sample size for this trial is 80. Thus, a total of 160 participants will be recruited.

## Statistical analysis

The principle of intention-to-treat (ITT) analysis will be followed in the process of statistical analysis. The last observation carried forward (LOCF) analysis will be applied for missing data in ITT analysis. Supplementary per-protocol analysis will also be carried out. All the statistical analyses will be performed using SPSS Ver. 24.0 with a significance level of 0.05.

First, the different baseline characteristics of the two groups will be described, and data will be presented as mean or median with standard deviation or interquartile range for continuous variables and as frequency distributions for categorical variables. Moreover, the histograms, boxes, and other representations will also be considered for the graphic analysis of corresponding data. A two-sample *t*-test for quantitative data or a chi-squared test for qualitative data will be performed as a homogeneity test, and the covariance analysis will also be performed if an adjustment is needed for a baseline characteristic.

Second, the analyses will focus on whether statistically better treatment outcomes could be achieved in the intervention group. *P*-values less than 0.05 will be considered statistically significant, and tests will be two-sided.

In terms of the changes in the measurement data, the two-sample *t*-test or the Wilcoxon rank-sum test will be used to compare the two groups and to determine differences according to normality. The mean change and the interaction between groups and observed time frames will be analyzed using a two-factor repeated measure analysis. If necessary, a mixed model approach will also be used.

Finally, safety analysis will be performed using the chi-squared test or Fisher's exact test based on the frequency and percentage of all recorded adverse events between the two groups.

The above plan is what we intend to do; however, the final analyses reported may differ from those planned, allowing for *post-hoc* analysis where it is indicated.

## Discussion

Based on the conception of ERAS, the treatment is designed to start before surgery to reduce the incidence of POI, which happens to have the same view as traditional Chinese medicine, “treat before disease attacks.” In addition, the study also set the ERAS group as the contrast, which may relatively objectively assess the effectiveness and safety of the two treatments.

As acupuncture has been widely accepted and applied in various diseases worldwide, other traditional Chinese therapies can also be taken into consideration, such as auricular acupressure. Auricular acupressure is low-cost, non-invasive, and has been approved for effectiveness in the digestive system (34). In clinical treatment, these two therapies are always combined to treat digestive diseases; however, few research studies have focused on such comprehensive therapy. Therefore, this study is supposed to approve the comprehensive effect of manual acupuncture combined with auricular acupressure in treating postoperative ileus.

The limitation of the study is that due to the specialization of acupuncture manipulation, double-blindness is hard to perform, so assessor-blindness is designed to decrease bias, and we have not set the sham-acupuncture group for the consideration of acceptability of postoperative patients.

To summarize, this trial aims to investigate the efficacy and safety of manual acupuncture combined with auricular acupressure in improving the symptoms and reducing the incidence of postoperative ileus. The results will be revealed at the end of the study and are expected to provide an alternative treatment option for preoperative patients, as well as contribute to the development of enhanced recovery after surgery.

## Trial status

The study is currently in the recruitment phase. The first patient was enrolled in November 2023, and the study is expected to end in March 2026.
